# Phase II study of capecitabine plus oxaliplatin (CapOX) as adjuvant chemotherapy for locally advanced rectal cancer (CORONA II)

**DOI:** 10.1007/s10147-019-01546-3

**Published:** 2019-09-21

**Authors:** Norifumi Hattori, Goro Nakayama, Keisuke Uehara, Toshisada Aiba, Kiyoshi Ishigure, Eiji Sakamoto, Yuichiro Tojima, Mitsuro Kanda, Daisuke Kobayashi, Chie Tanaka, Suguru Yamada, Masahiko Koike, Michitaka Fujiwara, Masato Nagino, Yasuhiro Kodera

**Affiliations:** 1 Department of Gastroenterological Surgery, Nagoya University Graduate School of Medicine, 65 Tsurumai-cho, Showa-ku, Nagoya, Aichi 466-8550 Japan; 2 Department of Surgical Oncology, Nagoya University Graduate School of Medicine, Nagoya, Japan; 3 Department of Surgery, Konan Kosei Hospital, Konan, Japan; 4 Department of Surgery, Nagoya Daini Red Cross Hospital, Nagoya, Japan; 5 Department of Surgery, Chukyo Hospital, Nagoya, Japan

## Abstract

**Objective:**

This multicenter, single-arm phase II study (UMIN000008429) aimed to evaluate the efficacy and safety of capecitabine plus oxaliplatin (CapOX) as postoperative adjuvant chemotherapy for patients with locally advanced rectal cancer.

**Methods:**

Patients with resectable clinical Stage II or III rectal cancer were enrolled to receive eight cycles of CapOX therapy (130 mg/m^2^ oxaliplatin on day 1 and 2000 mg/m^2^ oral capecitabine on days 1–14, every 3 weeks) after curative surgical resection. The primary endpoint was 3-year relapse-free survival (RFS) rate, and secondary endpoints were 3-year overall survival (OS) rate, treatment compliance, and safety.

**Results:**

A total of 40 patients (Stage II, 21; Stage III, 19) were enrolled between September 2012 and November 2015 from seven institutions. Thirty-nine patients (97%) received R0 resection, and 32 patients (84%) received postoperative CapOX therapy. The completion rate of all eight cycles of CapOX therapy was 66%. Relative dose intensities were 87% for oxaliplatin and 84% for capecitabine. At a median follow-up period of 46 months, disease recurrence was observed in nine patients, including three with local recurrence. Three-year RFS and OS rates were 75% (95% CI 57–86%) and 96% (95% CI 80–99%), respectively. Frequencies of Grade ≥ 3 hematological and non-hematologic adverse events were 19% and 38%, respectively.

**Conclusion:**

CapOX therapy is feasible as adjuvant chemotherapy for locally advanced rectal cancer.

## Introduction

Colorectal cancer is one of the leading causes of cancer death, and rectal cancer represents about 40% of all colorectal cancers [1]. The standard treatment for locally limited disease has been resection of the primary lesion with adequate lymphadenectomy. In Japan, a combination of surgery including lateral lymph node dissection and postoperative adjuvant chemotherapy represents the standard treatment of resectable locally advanced rectal cancer [2]. On the other hand, preoperative CRT for advanced lower rectal cancer is commonly performed in Europe and the United States [3-6], and few reports exist on the outcomes of treatment comprising postoperative adjuvant chemotherapy alone. For colon cancer, surgical resection followed by adjuvant chemotherapy with fluoropyrimidine has been established as the standard treatment for locally advanced disease [7-13]. Furthermore, several randomized controlled trials have shown that adjuvant chemotherapy with oxaliplatin in combination with fluoropyrimidine improves the survival of patients with resected Stage III colon cancer [9-11].

Clinical evidence on the efficacy of adjuvant chemotherapy for rectal cancer is relatively limited compared with those for colon cancer. The NSAS-CC trial showed that adjuvant chemotherapy with UFT, an oral fluoropyrimidine, following curative resection for Stage III rectal cancer had a survival benefit compared with surgery alone [14]. The ACTS-RC trial, which compared UFT with S-1 for Stage II/III rectal cancer, showed that S-1 therapy was superior to UFT with respect to recurrence-free survival time [15]. However, the efficacy and safety of oxaliplatin-based adjuvant chemotherapy for locally advanced rectal cancer remain unclear.

Thus, this multicenter, single-arm phase II study aimed to examine the efficacy and safety of capecitabine plus oxaliplatin (CapOX) as postoperative adjuvant chemotherapy in Japanese patients with locally advanced rectal cancer.

## Patients and methods

### Study design

This multicenter, single-arm phase II clinical trial was conducted by the Chubu Clinical Oncology Group (CCOG) and Nagoya Society of Oncology Group (NSOG) at seven hospitals in Japan. Inclusion criteria were as follows: age ≥ 20 years, histologically proven rectal adenocarcinoma located below the peritoneal reflection, locally advanced rectal cancer with clinical T3–4 tumor or positive lymph nodes, lack of distant metastasis, no prior chemotherapy and radiotherapy, Eastern Cooperative Oncology Group (ECOG) performance status of 0 or 1, and adequate hematologic, hepatic, and renal function. Patients with clinically significant cardiovascular disease, double cancer, bowel obstruction, Grade ≥ 1 peripheral sensory neuropathy (PSN), or uncontrolled diabetes mellitus were excluded.

This study was conducted in accordance with the principles set forth in the Declaration of Helsinki. Patients provided written informed consent prior to participation. The ethics committees of Nagoya University Hospital (approval number 2014-0043) and all other participating facilities approved the study. This trial was registered with the University Hospital Medical Information Network (UMIN000008429).

### Treatment plan

Patients were enrolled within 2 weeks before surgery, which included total mesorectal excision (TME) or tumor-specific mesorectal excision (TSME) with adequate lymphadenectomy according to Japanese guidelines for the treatment of colorectal cancer. For patients who required extended resection to achieve *R*0 curative resection, combined resection of the autonomic nerve, pelvic vessels, and adjacent organs was performed at the surgeons’ discretion. As adjuvant chemotherapy, CapOX therapy, which consisted of intravenous infusion of 130 mg/m^2^ oxaliplatin on day 1, along with oral administration of 2000 mg/m^2^ capecitabine on days 1–14, repeated every 3 weeks, was initiated in patients with pathological T3–4 tumors or positive lymph nodes within 8 weeks after surgery. Treatment was continued until completion of eight cycles or any one of the following occurred: disease progression, unacceptable toxicity, deterioration of ECOG performance status to > 2, or withdrawal of patient consent. Dose modification in response to treatment-related toxicities was carried out in accordance with the study protocol.

### Endpoints

The primary endpoint was 3-year relapse-free survival (RFS) rate, with RFS defined as the time from surgery until disease recurrence or death from any cause. Secondary endpoints included 3-year overall survival (OS) rate, with OS defined as the time from surgery until death from any cause; cumulative local and distant recurrence rates; *R*0 resection rate; treatment compliance; and adverse events. Recurrence was assessed by computed tomography every 6 months after surgery and total colonoscopy once every year. Adverse events were graded according to the National Cancer Institute Common Terminology Criteria (NCI-CTC), version 3.0.

All analyses of efficacy were based on the intent-to-treat (ITT) population, defined as eligible and assessable enrolled patients. The safety population was defined as all patients receiving ≥ 1 dose of the protocol treatment.

### Statistical analysis

Statistical power was calculated based on the following assumptions: 3-year RFS rate threshold of 50% and an expected 3-year RFS rate of 70% based on data from previous clinical trials [14], with an enrollment period of 2 years and follow-up period of 5 years. To ensure an alpha level of 0.05 (one-side) and a detection power (1 − β) of 80%, 37 patients were required. Therefore, the planned sample size was 40 patients to account for possible loss to follow-up. Differences in characteristics between the two arms were analyzed using the *χ*2 test for categorical variables and the Mann–Whitney *U* test for continuous variables. Time-to-event variables, RFS, and OS were analyzed by the Kaplan–Meier method. *P* values less than 0.05 were considered statistically significant. Statistical analyses were performed using JMP software version 13 (SAS Institute Inc., Cary, NC, USA).

## Results

### Patient characteristics

**Fig. 1:**
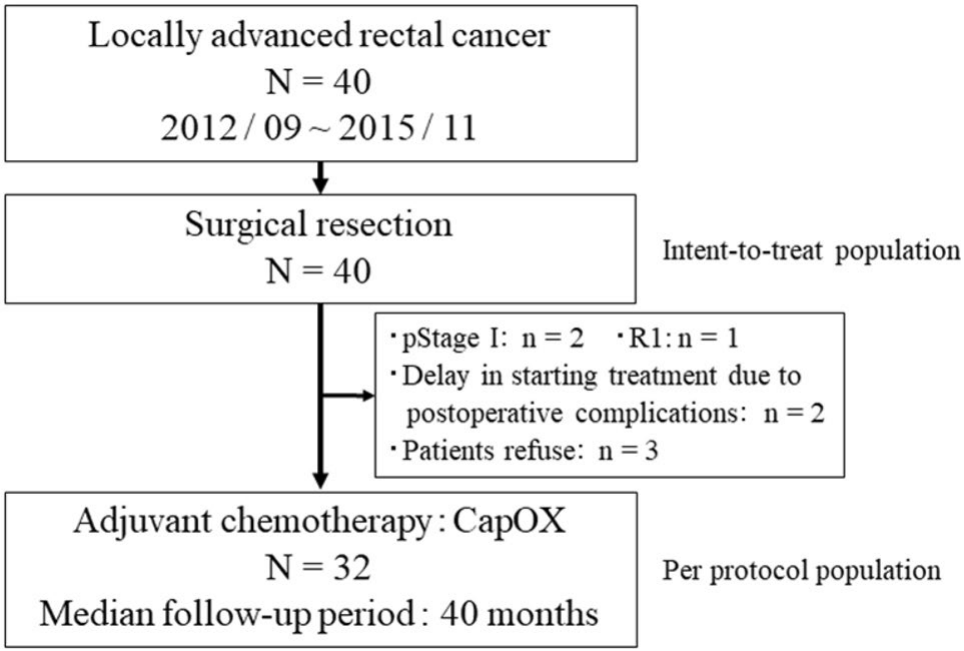
Consort diagram. A total of 40 patients enrolled between September 2012 and November 2015 underwent surgical resection. CapOX adjuvant chemotherapy was administered to 32 patients. *N* total number of patients, *n* number of patients, *CapOX* capecitabine and oxaliplatin

A total of 40 patients were prospectively enrolled between September 2012 and November 2015 from 7 institutions. The ITT population comprised 40 patients. Eight patients were excluded from the safety analysis for the following reasons: two were diagnosed with pathological Stage I disease: (1) received non-curative resection, (2) had postoperative complications, and (3) refused protocol treatment. Thus, the safety population comprised 32 patients (Fig. 1). Baseline clinical characteristics are summarized in Table 1. The relationship between clinical and pathological staging is shown in Table 2. The agreement between clinical and pathological TN factors was 60% (24/40) and 67.5% (27/40), respectively. The correlation between cStage and pStage resulted in an agreement of 75% (30/40).

**Table 1:** Patient characteristics

Variable	*N*=40		
	*n*		%
Gender			
Male	25		63
Female	15		37
Age years			
Median (range)		68 (49–80)	
PS (ECOG)			
0	35		88
1	5		12
BMI			
Median (range)		21.5 (13.5–31.4)	
Tumor diameter (mm)			
Median (range)		40 (20–80)	
Distance from anal verge to tumor (cm)			
Median (range)		5.5 (0.5–15)	
cT			
T2	4		10
T3	24		60
T4	12		30
cN			
*N*0	21		53
*N*1	14		35
*N*2	5		12
cStage			
II	21		53
III	19		47
Pathology			
Tub	38		95
por/muc	2		5
Operative procedure			
Low anterior resection	29		73
Abdominal peritoneal resection	9		23
Hartmann operation	1		2
Intersphincteric resection	1		2
Approach			
Open	13		33
Laparoscopy	27		67
Lateral lymph node dissection			
Bilateral	7		18
Unilateral	2		4
None	31		78

*N* total number of patients, *n* number of patients, *PS* performance status, *ECOG* Eastern Cooperative Oncology Group, *BMI* body mass index

**Table 2:** Relationship between clinical and pathological staging

	pTl	pT2	pT3	pT4
cT2 (*n* = 4)	1	1	2	
cT3 (*n* = 24)		2	19	3
cT4 (*n* = 12)		1	7	4

	pN0	pNl	pN2	pN3
cN0 (*n* = 21)	17	3	1	
cNl (*n* = 14)	4	7	3	
cN2 (*n* = 5)		1	3	1
cN3 (*n* = 0)				

	pStage I	pStage II	pStage III
cStage II (*n* = 21)	2	15	4
cStage III (*n* = 19)		4	15

*n* number of patients

### Surgical procedures

All 40 patients received surgical procedures, including low anterior resection with TME in 20 patients and with TSME in nine patients, and Miles’ operation in nine patients. *R*0 resection was achieved in 39 patients (98%). The median number of dissected lymph nodes was 22 (range 6–47). Postoperative complications (Clavien–Dindo classification Grade 2 and higher) were observed in 13 patients (33%), including surgical site infection in four, ileus in six, anastomotic leakage in three, and dysuria in three.

### Treatment exposure

Thirty-two patients (82%) were treated with CapOX therapy. The median time from surgery to initiation of adjuvant chemotherapy was 43 days (range 10–56 days). The median number of cycles was eight (range 1–8 cycles), and the overall treatment completion rate was 66%. The relative dose intensity was 87% (range 12–100%) for oxaliplatin and 84% (range 12.5–100%) for capecitabine (Table 3).

**Table 3:** Adjuvant treatment status

	*N* = 32
Time from surgery to initiation of chemotherapy (days)	
Median (range)	43(10–56)
Treatment cycles received	
Median (range)	8(1–8)
Completion rate (%)	66%
RDI (%)	
Capecitabine	
Median (range)	84(12.5–100)
Oxaliplatin	
Median (range)	87(12–100)

*N* total number of patients, *RDI* relative dose intensity

### Efficacy

**Fig. 2:**
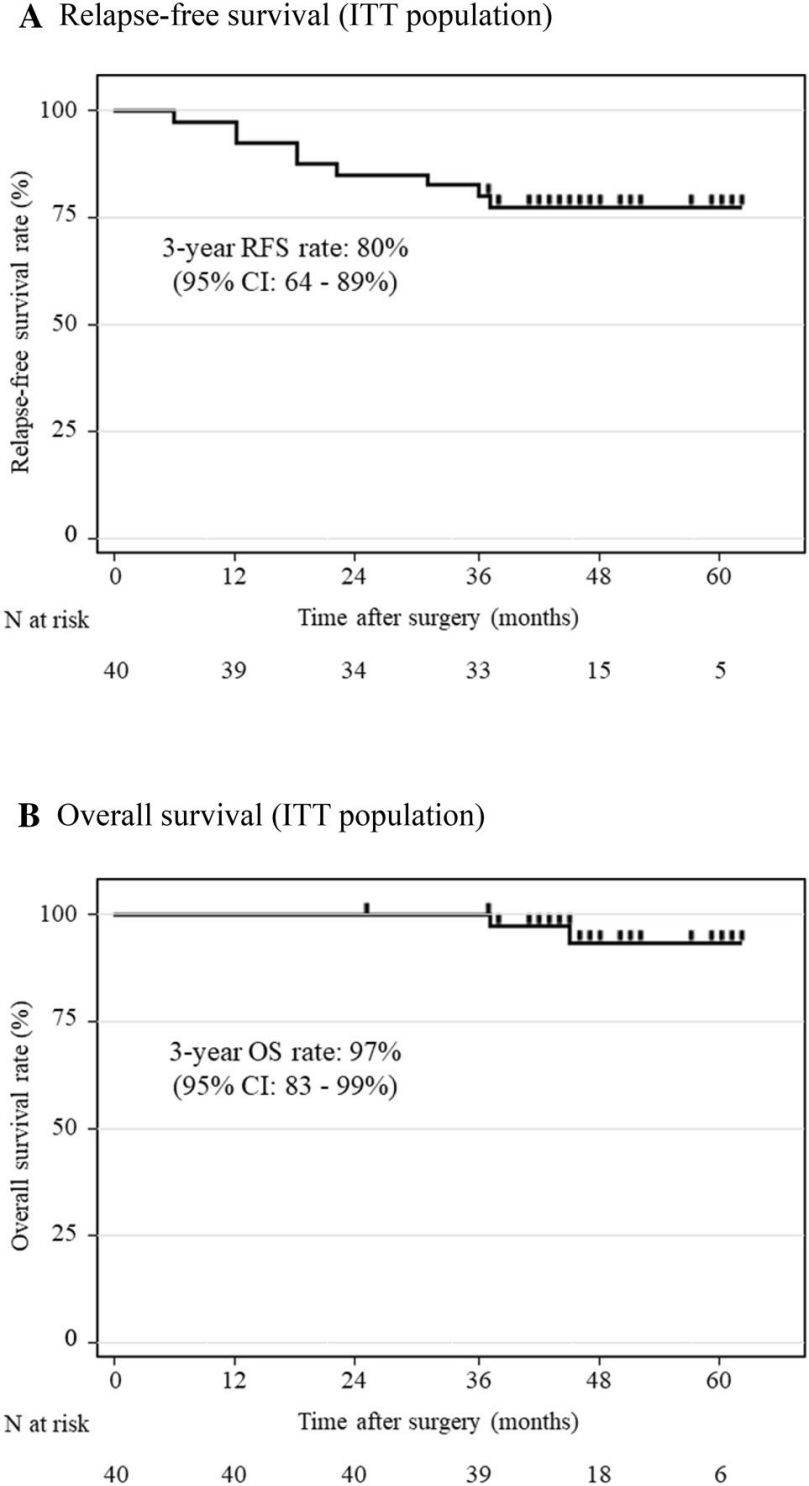
Survival outcomes (ITT population). **a** Three-year RFS rate was 80% (95% CI 64–89%). **b** Three-year OS rate was 97% (95% CI 83–99%). *RFS* relapse-free survival, *OS* overall survival, *N* total number of patients

**Fig. 3:**
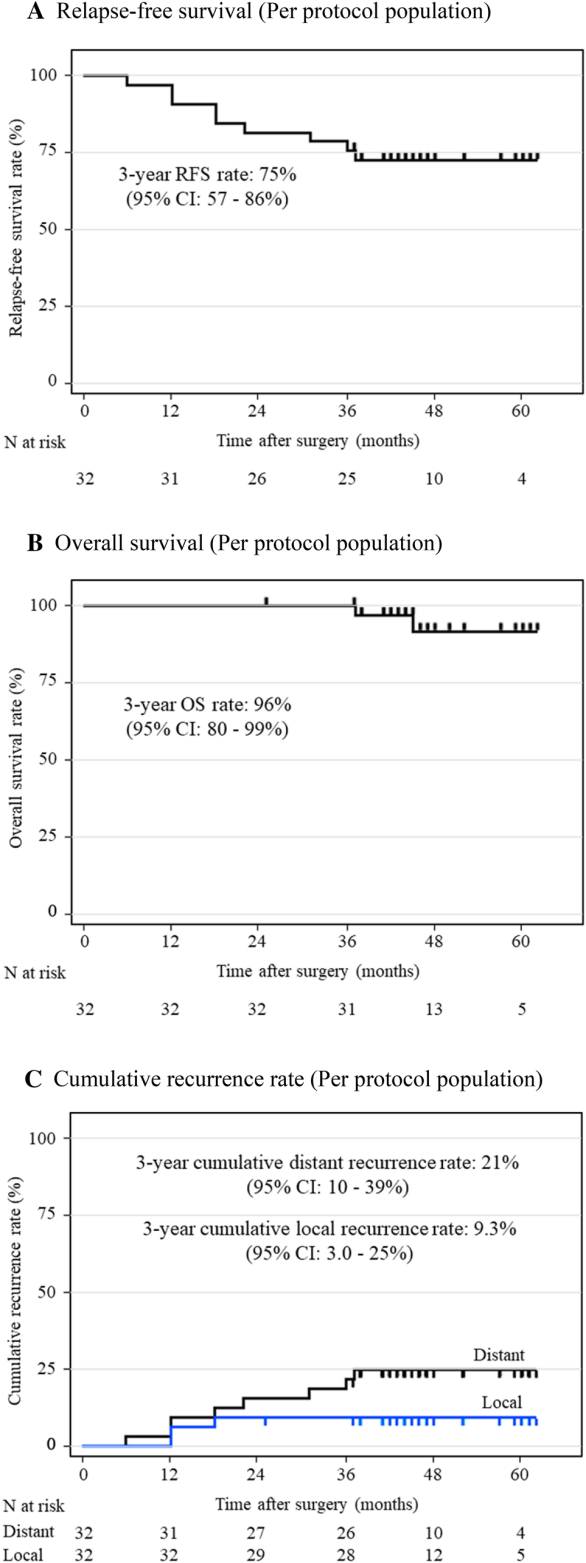
Survival outcomes (per protocol population). **a** Three-year RFS rate was 75% (95% CI 57–86%). **b** Three-year OS rate was 96% (95% CI 80–99%). **c** Three-year cumulative rate of local recurrence and distant recurrence were 9.3% (95% CI 3.0–25%) and 21% (95% CI 10–39%), respectively. *RFS* relapse-free survival, *OS* overall survival, *N* total number of patients

Data cutoff for the final analysis was set as December 1, 2018. The median length of follow-up for censored cases was 46 months (range 25–62 months). Disease recurrence after adjuvant chemotherapy was observed in nine patients (28%), including three with local recurrence and eight with distant recurrence (Table 4). The 3-year RFS rate (primary endpoint) was 80% (95% CI 64–89%) (Fig. 2a). The 3-year OS rate was 97% (95% CI 83–99%) (Fig. 2b). In the per protocol population, 3-year RFS and OS rates were 75% (95% CI 57–86%) and 96% (95% CI 80–99%), respectively (Fig. 3a, b). In the prognosis of each staging, the 3-year RFS rate was 93% (95% CI 64–99%) in pStage II and 52% (95% CI 30–74%) in pStage III, and the 3-year OS rate was 100% in pStage II and 93% (95% CI 66–99%) in pStage III. Three-year cumulative rates for local recurrence and distant recurrence were 9.3% (95% CI 3.0–25%) and 21% (95% CI 10–39%), respectively (Fig. 3c).

**Table 4:** Recurrence pattern

	*N* = 32	
	*n*	%
Recurrence	9	28
Local recurrence	3	9
Anastomotic	1	
Pelvic	2	
Distant recurrence	8	25
Liver	1	
Lung	2	
Para-aortic lymph node	5	

*N* total number of patients, *n* number of patients

### Safety

The incidence of treatment-related adverse events is shown in Table 5. Frequencies of Grade ≥ 3 hematologic and non-hematologic adverse events were 19% and 38%, respectively. The most frequent Grade ≥ 3 hematologic and non-hematologic adverse events were thrombocytopenia (12.5%) and PSN (15.6%), respectively. The incidence of toxicity-related treatment discontinuation was 73%, and the most common adverse event that led to discontinuation was PSN (38%). PSN lasting after the end of adjuvant treatment was experienced by eight patients. No patient had persistent Grade ≥ 3 PSN.

**Table 5:** Frequency of common toxicities

	*N* = 32			
	All grade		≥ Grade 3	
	*n*	%	*n*	%
Hematologic toxicity	21	65.6	6	19
Neutropenia	17	53.1	3	9.3
Anemia	21	65.6	1	3.1
Thrombocytopema	21	65.6	4	12.5
Febrile neutropenia	0	0	0	0
Non-hematologic toxicity	26	81.2	12	38
Anorexia	18	56.2	4	12.5
Diarrhea	8	25	1	3.1
Nausea/vomiting	17	53.1	3	9.3
Mucositis	5	15.6	0	0
Hand-foot syndrome	11	34.3	1	3.1
Peripheral neuropathy	26	81.2	5	15.6
Allergy	3	9.3	1	3.1

## Discussion

This phase II trial was conducted to evaluate the efficacy and safety of CapOX as postoperative adjuvant chemotherapy for Stage II/III rectal cancer. The 3-year RFS rate (primary endpoint) of 80% was considered acceptable according to our hypothesis that assumes threshold and expected survival rates of 50% and 70%, respectively. Furthermore, the 3-year RFS and OS rate (per protocol population) were 75% and 96%, comparable to those reported in two RCTs evaluating adjuvant chemotherapy for resectable rectal cancer without preoperative therapy [14, 15].

Multimodal therapy comprising preoperative fluoropyrimidine with concurrent radiotherapy followed by TME and adjuvant fluoropyrimidine-based chemotherapy is recommended as a global standard for patients with Stage II/III rectal cancer [16, 17]. The reported 3-year RFS and local recurrence rates of neoadjuvant CRT plus adjuvant chemotherapy for patients with locally advanced rectal cancer range from 62.9 to 72.7% and 4.4 to12.1%, respectively [18-20]. Although we could not directly compare prognosis between patients who received postoperative adjuvant chemotherapy alone and neoadjuvant CRT plus adjuvant chemotherapy, the 3-year RFS and local recurrence rates in our study were comparable to treatment outcomes for neoadjuvant CRT plus adjuvant chemotherapy. Surprisingly, these favorable outcomes were achieved by adjuvant chemotherapy alone following curative resection.

The benefits of oxaliplatin-based neoadjuvant CRT and adjuvant chemotherapy in Stage II/III rectal cancer are controversial. At least seven randomized trials [18–24] have investigated the effects of oxaliplatin-based neoadjuvant CRT and adjuvant chemotherapy in Stage II/III rectal cancer [25]. Only the ADORE randomized phase II study showed that adjuvant FOLFOX improved disease-free survival compared with fluorouracil plus leucovorin in patients with locally advanced rectal cancer after preoperative CRT and total mesorectal excision [19]. The 3-year RFS and OS rates in the present trial were comparable to those of NSAS-CC and ACTS-RC trials which used an oral fluoropyrimidine alone as adjuvant chemotherapy. The reason for not showing superiority of adding oxaliplatin to fluoropyrimidine may involve differences in patient characteristics. In the present trial, the main tumor was located in the rectum with the lower margin below the peritoneal reflection and the proportion of patients with T4 tumors was 30%. However, the NSAS-CC trial included patients with RS tumors, and only 10% of patients had T4 tumors in the ACTS-RC trial. To demonstrate the benefit of adding oxaliplatin to fluoropyrimidine, a study comparing oral fluoropyrimidine monotherapy with a combination therapy comprising oxaliplatin and oral fluoropyrimidine as adjuvant chemotherapy for rectal cancer will be needed.

Surgical treatment outcomes in our cohort were favorable. The *R*0 resection rate was 97.5% and the rate of anastomotic leakage was 10%. With regard to neoadjuvant therapy for locally advanced rectal cancer, a major issue is the increase in complications of curative surgery with preoperative therapy. According to a recent clinical trial, CRT increased perioperative complications such as anastomotic leakage and surgical site infection [24]. Moreover, the rate of anastomotic leakage in the present trial was lower than that reported in a phase II trial of perioperative CapOX without radiotherapy for high-risk rectal cancer (CORONA I study) [26].

The MOSAIC, NSABPC-07, and NO16968 trials have demonstrated the superiority of adding oxaliplatin to fluoropyrimidine in the adjuvant setting for Stage III colon cancer, irrespective of how fluoropyrimidine is delivered (e.g., intravenous infusion, bolus, or oral) [9–11]. The present trial used an oral fluoropyrimidine (capecitabine), which is potentially more convenient for patients and oncologists compared with intravenous 5-FU-based regimens. Kopec et al. [27] reported that patients who received adjuvant therapy with oral fluoropyrimidine perceived it to be more convenient than intravenous 5-FU. We believe that CapOX reduces medical resource use (e.g., drug administration visits, central venous port placement, and removal) and places less of a time burden on patients compared with FOLFOX.

In terms of safety, the most frequent adverse events associated with adjuvant CapOX therapy were PSN and hematologic events, such as anemia and thrombocytopenia. Of Grade ≥ 3 adverse events, frequencies for PSN, neutropenia, and thrombocytopenia were 15.6%, 9.3%, and 12.5%, respectively. These findings are consistent with those of the NO16968 trial [28]. PSN resulting from the use of oxaliplatin is a cumulative dose-related toxicity. In the present trial, 25% of patients had persistent Grade 1/2 neurosensory toxicity at 12 months after the end of adjuvant treatment. Pachman et al. reported that about 30% of patients experienced moderate neuropathy as measured by the European Organisation for Research and Treatment of Cancer Quality of Life Questionnaire for patients with chemotherapy-induced peripheral neuropathy (EORTC-CIPN) and about 20% of patients experienced severe neuropathy at 18 months from adjuvant oxaliplatin therapy [29]. Thus, a shorter duration of adjuvant CapOX therapy would be beneficial so long as efficacy is not affected. In this regard, the ACHIEVE trial confirmed that a shorter duration of oxaliplatin-based adjuvant chemotherapy resulted in a significant reduction of PSN in a Japanese population with Stage III colon cancer [30].

This trial has a number of limitations worth noting. First, the single-arm design and relatively small sample size necessitate confirmation of our results in a larger cohort. Second, the numbers of cases with bulky tumors > 10 cm, with T4, and that were lymph node-positive were fewer than those of the CORONA I study. It will be necessary to carefully consider curative resection with adjuvant chemotherapy alone for all Stage II/III rectal cancer cases and to consider treatment strategies including perioperative chemotherapy based on the state of tumor progression.

In conclusion, we found that CapOX as adjuvant chemotherapy for locally advanced rectal cancer is a feasible regimen. CapOX may serve as an important option for patients who have not undergone preoperative therapy.
